# Knowledge-based extraction of adverse drug events from biomedical text

**DOI:** 10.1186/1471-2105-15-64

**Published:** 2014-03-04

**Authors:** Ning Kang, Bharat Singh, Chinh Bui, Zubair Afzal, Erik M van Mulligen, Jan A Kors

**Affiliations:** 1 Department of Medical Informatics, Erasmus University Medical Center, P.O. Box 2040, 3000, CA, Rotterdam, The Netherlands

**Keywords:** Relation extraction, Knowledge base, Adverse drug effect

## Abstract

**Background:**

Many biomedical relation extraction systems are machine-learning based and have to be trained on large annotated corpora that are expensive and cumbersome to construct. We developed a knowledge-based relation extraction system that requires minimal training data, and applied the system for the extraction of adverse drug events from biomedical text. The system consists of a concept recognition module that identifies drugs and adverse effects in sentences, and a knowledge-base module that establishes whether a relation exists between the recognized concepts. The knowledge base was filled with information from the Unified Medical Language System. The performance of the system was evaluated on the ADE corpus, consisting of 1644 abstracts with manually annotated adverse drug events. Fifty abstracts were used for training, the remaining abstracts were used for testing.

**Results:**

The knowledge-based system obtained an F-score of 50.5%, which was 34.4 percentage points better than the co-occurrence baseline. Increasing the training set to 400 abstracts improved the F-score to 54.3%. When the system was compared with a machine-learning system, jSRE, on a subset of the sentences in the ADE corpus, our knowledge-based system achieved an F-score that is 7 percentage points higher than the F-score of jSRE trained on 50 abstracts, and still 2 percentage points higher than jSRE trained on 90% of the corpus.

**Conclusion:**

A knowledge-based approach can be successfully used to extract adverse drug events from biomedical text without need for a large training set. Whether use of a knowledge base is equally advantageous for other biomedical relation-extraction tasks remains to be investigated.

## Background

Vast amounts of biomedical information are only offered in unstructured form through scientific publications. It is impossible for researchers or curators of biomedical databases to keep pace with all information in the growing number of papers that are being published [1,2]. Text-mining systems hold promise for facilitating the time-consuming and expensive manual information extraction process [3], or for automatically engendering new hypotheses and fresh insights [4,5].

In recent years, many systems have been developed for the automatic extraction of biomedical events from text, such as protein-protein interactions and gene-disease relations [2,6]. Relatively few studies addressed the extraction of drug-related adverse effects, information which is relevant in drug research and development, healthcare, and pharmacovigilance [7]. The reason that this subject has been studied less frequently may in part be explained by the scarcity of large annotated training corpora. Admittedly cumbersome and expensive to construct, these data sets are nonetheless essential to train the machine-learning based classifiers of most current event extraction systems. Relation extraction systems typically perform two tasks: first, they try to recognize the entities of interest, next they determine whether there are relations between the recognized entities. In many previous studies, system performance evaluation was often limited to the second, relation extraction task, and did not consider the performance of the entity recognition task.

In this study, we describe the use of a knowledge base to extract drug-adverse effect relations from biomedical abstracts. The main advantage of our system is that it needs very little training data as compared to machine-learning approaches. Also, we evaluate the performance of the whole relation extraction pipeline, including the entity recognition part.

### Related work

To extract biomedical relations from unstructured text a number of approaches have been explored, of which we mention simple co-occurrence, rule-based, and machine-learning based techniques.

The simplest approach is based on the co-occurrence of entities of interest. It assumes that if two entities are mentioned together in the same sentence or abstract, they are probably related. Typically, this approach achieves high recall, but low precision [8]. Since co-occurrence approaches are straightforward and do not involve linguistic analysis, their performance is often taken as a baseline to gauge other methods [9,10].

Rule-based techniques are also a popular method for relation extraction. The rules are defined manually using features from the context in which the relations of interest occur. Such features may be prefixes and suffixes of words, part-of-speech (POS) tags, chunking information, etc. [11-13]. However, the large amount of name variations and ambiguous terms in the text may cause an accumulation of rules [5]. This approach can increase precision, but often at the cost of significantly lower recall [14].

Machine-learning approaches automatically build classifiers for relation extraction, using contextual features derived from natural language processing techniques such as shallow parsing, which divides the sentence into chunks [15,16], or full dependency parsing, which provides a complete syntactic analysis of sentence structures [17]. The performance of these methods is usually good [18-20], but they require annotated training sets of sufficient size. Also, processing time may be high [3].

Hybrid approaches that combine manual and automatic approaches have also become more popular in recent years [21,22].

An example of a relation extraction system is JReX, developed by the JULIE lab [23]. JReX uses a support vector machine (SVM) algorithm as its classifier. Originally developed for the extraction of protein-protein interactions, it was later adapted to the domain of pharmacogenomics. Using the PharmGKB database [24], JReX obtained F-scores in the 80% range for gene-disease, gene-drug, and drug-disease relations [25]. The Semantic Knowledge Representation (SKR) system [26], developed by the National Library of Medicine, provides semantic representations of biomedical text by building on resources currently available at the library. SKR applies two programs, MetaMap [27] and SemRep [28], both of which utilize information available in the Unified Medical Language System (UMLS) [29]. SKR has been used for concept-based query expansion, for identification of anatomical terminology and relations in clinical records, and for mining biomedical texts for drug-disease relations and molecular biology information [30]. Java Simple Relation Extraction (jSRE) is still another relation extraction tool based on SVM. It has been used for the identification and extraction of drug-related adverse effects from Medline case reports [31,32], achieving an F-score of 87% on the ADE corpus [33]. It should be noted that this high performance value was obtained on a selected set of sentences that contained relatively many drug-adverse event relations. A framework that integrates nine event extraction systems is U-Compare [34]. The U-Compare event meta-service provides an ensemble approach to relation extraction, where the combination of systems may produce a significantly better result than the best individual system included in the ensemble [34]. Hybrid approaches that combine different techniques have also been shown to perform well. Bui et al. [35] proposed a novel, very fast system that combines natural language processing (NLP) techniques with automatically and manually generated rules, and obtained an F-score of 53% on the Genia event corpus [36], a result that is comparable to other state-of-the-art event extraction systems.

Most of the existing relation extraction systems use machine-learning algorithms and require an annotated corpus for training. There are several publicly available biomedical text corpora with manually annotated relations, for instance the corpora generated as part of the Biocreative [37-39] and BioNLP [40,41] challenges, the GENIA event corpus [36], PharmGKB [24], and the ADE corpus [33]. Most of these corpora focus on protein-protein interactions or other bio-events, while only two address drug-disease relations (PharmGKB) or drug-adverse effect relations (ADE corpus). As some of the annotations in PharmGKB have been reported to be hypothetical [42], we chose to use the ADE corpus as the gold standard corpus (GSC) for our experiments.

## Methods

### Corpus

The ADE corpus is originally based on 2972 Medline abstracts of case reports that were manually annotated for adverse drug effects [33]. The case reports were selected by a PubMed query with the MeSH (Medical Subject Headings) terms “drug therapy” and “adverse effect”. Only the sentences that contain at least one adverse drug effect have been made available by the corpus developers. The ADE corpus consists of 4272 of these sentences, taken from 1644 abstracts. The sentences contain annotations of 5063 drugs, 5776 conditions (diseases, signs, symptoms), and 6821 relations between drugs and conditions representing clear adverse effect occurrences [33]. Each relation consists of a Medline identifier, the sentence that contains this relation, the text and position of the drug, and the text and position of the adverse effect. Relations were only annotated if they occur in a single sentence. Drugs and conditions were not annotated if they were not part of an adverse event relation. We divided the 1644 abstracts that have sentences in the ADE corpus, into two sets: a small training set of 50 randomly selected abstracts, and a test set with the remaining abstracts (Table 1). Contrary to previous studies [32], we used all sentences in the 1644 abstracts, both the 4272 “positive” sentences that contain at least one relation according to the gold standard, and 7560 “negative” sentences that do not contain a relation.

**Table 1:** Number of abstracts, relations, and sentences in the ADE corpus

	**Training set**	**Test set**	**Total**
Abstracts	50	1594	1644
Relations	201	6620	6821
Sentences with at least one relation	130	4142	4272
Sentences with no relation	233	7327	7560

### Relation extraction system

The relation extraction system consists of two main modules: a concept identification module that identifies drugs and adverse effects, and a knowledge-base module that determines whether an adverse effect relation can be established between the entities that are found. All modules were integrated in the Unstructured Information Management Architecture framework [43].

We used the Peregrine system (https://trac.nbic.nl/data-mining/) as the basis of our concept identification system. Peregrine is a dictionary-based concept recognition and normalization tool, developed at the Erasmus University Medical Center [44]. It finds concepts by dictionary look-up, performs word-sense disambiguation if necessary, and assigns concept unique identifiers (CUIs). We used Peregrine with a dictionary based on version 2012AA of the UMLS Metathesaurus, only keeping concepts that belong to the semantic groups “Chemicals & Drugs” and “Disorders” [45]. Rewrite and suppress rules are applied to the terms in the dictionary to enhance precision and recall [46].

To further improve concept identification, we employed a rule-based NLP module that we previously developed and tested for disease identification [47]. Briefly, the NLP module consists of a number of rules that are divided into five submodules, which carry out coordination resolution, abbreviation expansion, term variation, boundary correction, and concept filtering. The rules combine the annotations of a concept normalization system, such as Peregrine, with POS and chunking information. The coordination module uses POS and chunking information to reformat the coordination phrase and feed the reformatted text into the concept normalization system for proper annotation of the concepts. The abbreviation module combines an abbreviation expansion algorithm [48] with POS and chunking information to improve the recognition of abbreviations. The term variation module contains a number of rules that adjust noun phrases and feed the adjusted phrase into the concept normalization system again, to check whether it refers to a concept. The boundary correction module contains several rules that correct the start- and end positions of concepts identified by the system, based on POS and chunking information. The concept filtering module consists of two rules that suppress concepts that were identified by the concept normalization system. One rule removes a concept if the concept annotation in the text has no overlap with a noun phrase because in our experience, most UMLS concepts in biomedical abstracts belong to a noun phrase, or at least overlap with it. The other rule removes a concept if it is part of a concept filter list. The NLP module was not modified for the current task except for the concept filter list, which was adjusted based on our training data.

The knowledge base is a graph representation of the information contained in the UMLS Metathesaurus and the UMLS Semantic Network. The UMLS Metathesaurus defines terms and concepts (CUIs), as well as relations between the concepts. Each relation has a relation type, e.g., “is-a” or “cause-of”. There are a total of 621 relation types in the UMLS Metathesaurus. The UMLS Semantic Network consists of a set of semantic types, i.e., broad subject categories that provide a categorization of all concepts represented in the UMLS Metathesaurus. The semantic types are connected by semantic relations.

The knowledge base is a three-tier hierarchical graph in which vertices represent terms, concepts, and semantic types, and the edges represent relations between concepts and between semantic types. At the lowest level are the terms, which are linked to concepts at the second level. Each concept is linked to one or more semantic types, which are situated at the highest level. The knowledge base has been implemented in a graph database (http://www.neo4j.org) and was populated with concepts (CUIs) and relations extracted from the UMLS 2012 AA release. In this study, we only used the relations at the second level, i.e., between concepts.

The edges that connect two concepts form a path, with a length equal to the number of edges. The distance between two concepts is defined as the length of the shortest path. Note that there may be multiple shortest paths, but there is only one shortest path length.

For each sentence in the corpus, we determined the distance in the knowledge base between the drugs and adverse effects that were found by the concept identification module. Only if the distance between a drug-adverse effect pair was less than or equal to a distance threshold, a relation was considered present. Based on our training set, we empirically found that a distance threshold of four gave best performance results.

Further reduction of false-positive drug-adverse effect relations was attempted by taking into account the type of the relations in the shortest paths between drugs and adverse events. In our training set, we counted the number of each relation type in the paths that resulted in false-positive and in true-positive drug-adverse effect relations. If for a relation type the ratio of the false-positive count plus one and the true-positive count plus one was greater than seven, we discarded any path containing that relation type. The value of seven was determined experimentally on the training set as yielding the best performance.

### Performance evaluation

In the ADE corpus, including both the 4272 positive and 7560 negative sentences, drug-adverse effect relations are annotated at the sentence level by specifying the start and end positions of the drug and the adverse effect. We counted a relation found by our system as true positive if the boundaries of the drug and adverse effect exactly matched those of the gold standard. If a gold-standard relation was not found, i.e., if the concept boundaries were not rendered exactly by the system, it was counted as false negative. If a relation was only found by the system, i.e., the concept boundaries did not exactly match the gold standard, it was counted as false positive. Performance was evaluated in terms of precision, recall, and F-score. An error analysis was carried out on a sample of 100 randomly selected errors that were made by our relation extraction system.

## Results

### Performance of the relation extraction system

Table 2 shows the performance of the Peregrine baseline system on the test set of the ADE corpus, and the incremental contribution for each of the different modules. The baseline system had a high recall but low precision, yielding an F-score of 16.1%. Use of the NLP module more than doubled the F-score. Application of the knowledge base further improved the F-score by 12.6 percentage points. Relation-type filtering increased the F-score by another 4.3 percentage points. Overall, the knowledge-base module decreased recall by 8.1 percentage points, but increased precision by 17.0 percentage points.

**Table 2:** Performance (in %) of the baseline relation extraction system and the incremental contribution of different system modules, on the test set of the ADE corpus

**System**	**Precision**	**Recall**	**F-score**
Baseline	8.9	78.4	16.1
+ NLP module	21.1	82.9	33.6
+ Knowledge base	32.8	78.1	46.2
+ Relation-type filtering	38.1	74.8	50.5

### Effect of different distance thresholds in the knowledge base

Table 3 shows the performance of the relation extraction system on the ADE test corpus for different distance thresholds (the maximum allowed length of the shortest path between a drug and an adverse effect) in the knowledge base. The highest F-score of 50.5% is obtained with a distance of four. Lowering the distance threshold increases precision and decreases recall. The highest recall is 76.5% (precision 37.0%) at a threshold of five, the highest precision is 43.2% (recall 1.6%) at a threshold of one.

**Table 3:** Performance (in %) of the relation extraction system on the test set of the ADE corpus for different distance thresholds in the knowledge base

**Threshold**	**Precision**	**Recall**	**F-score**
1	43.2	1.6	3.1
2	41.8	15.2	22.3
3	40.6	64.1	49.7
4	38.1	74.8	50.5
5	37.0	76.5	49.9

### Effect of different training set sizes

To assess the effect of increasing amounts of training data on system performance, training sets of 100, 200, and 400 abstracts were selected from the ADE corpus. The abstracts in a training set were a subset of the abstracts in the next larger training set. For each training set, the corresponding test set consisted of the remaining abstracts in the ADE corpus. Table 4 shows that the performance of the relation extraction system improves with larger amounts of training data, but is leveling off with increasing size. The system obtains an F-score of 54.3% when trained on 400 abstracts, which is an improvement of 3.8 percentage points as compared with the system trained on 50 abstracts The NLP module contributed 1.7 percentage points to this improvement, and the relation-type filter module 2.1 percentage points. The baseline Peregrine module and the knowledge-base module do not require training and thus were not changed.

**Table 4:** Performance (in %) of the relation extraction system on the test set of the ADE corpus for different sizes of the training set

**Abstracts for training**	**Precision**	**Recall**	**F-score**
50	38.1	74.8	50.5
100	39.8	75.2	52.1
200	41.1	75.7	53.3
400	42.1	76.3	54.3

### Performance comparison of knowledge based and machine-learning based relation extraction

Part of the ADE corpus that we used in our experiments, has previously been used by Gurulingappa et al. [32] to develop and evaluate a machine-learning based relation extraction system based on jSRE. To enable a comparison of the performance of our knowledge-based relation extraction system and the previously published results for jSRE, we set up the same training and test environment as described by Gurulingappa et al. [32]. Similar to Gurulingappa et al., we removed 120 relations with nested annotations in the gold standard (e.g., “acute lithium intoxicity”, where “lithium” is related to “acute intoxicity”), and only used the positive sentences in the ADE corpus. In [32], all remaining true relations (taken from the gold standard) were supplemented by false relations (taken from co-occurring drugs and conditions that were found by ProMiner [49], a dictionary-based entity recognition system), in a ratio of 1.26:1. To create a corpus with the same ratio to train and test our system and allow comparison of results, we took all true relations in which the concepts were found by Peregrine and the NLP module, and randomly added false co-occurrence relations generated by Peregrine and the NLP module, until the ratio of 1.26:1 was reached.

Table 5 shows the performance of our knowledge-base system and the previously reported performance of jSRE [32]. Without any training corpus, i.e., only applying the knowledge base but not the relation-type filtering, which requires training, our system already got an F-score of 88.5%. Additional use of the relation-type filter trained on small sets of 10 or 50 abstracts, resulted in slightly higher F-scores, which were substantially better than those obtained with jSRE. The best F-score reported for jSRE, when about 90% of the abstracts in the corpus was utilized for training, was 87% [32].

**Table 5:** **Performance (in %) of a machine-learning based (jSRE) relation extraction system [**[32]**] and the knowledge-based system on a subset of the ADE test corpus (see text)**

**Training set (abstracts)**	**Machine learning**			**Knowledge base**		
	**Precision**	**Recall**	**F-score**	**Precision**	**Recall**	**F-score**
0	n/a	n/a	n/a	88.5	88.6	88.5
10	58	6	55	89.1	88.2	88.6
50	79	87	82	91.8	86.1	88.8

### Error analysis

We randomly selected 100 errors that the system made in our test set, and manually classified them into different error types (Table 6). False-positive errors were mostly due to drugs and adverse effects that were correctly found by the concept identification module, but were wrongly annotated by the knowledge-base module as having a relation. Of the 64 errors of this type, 46 occurred in negative sentences, i.e., sentences that do not contain any drug-adverse effect relation according to the gold standard. For instance, the gold standard did not annotate a relation in “Norethisterone and gestational diabetes”, but the system found “norethisterone” as a drug concept, “gestational diabetes” as an adverse effect, and generated a false-positive relation between these two concepts. Eighteen of the 64 errors occurred in positive sentences. For instance, in the sentence “Pneumocystis carinii pneumonia as a complication of methotrexate treatment of asthma”, the gold standard annotated a relation between the drug “methotrexate” and the adverse effect “pneumocystis carinii pneumonia”, concepts that were also found by the system. However, the system also annotated “asthma” as another adverse effect concept, which generated a false-positive relation between “methotrexate” and “asthma”. The second type of false-positive errors was caused by incorrectly found concepts, for which a relation was found in the knowledge base. For instance, in “Drug-induced pemphigus related to angiotensin-converting enzyme inhibitors”, the system incorrectly annotated “angiotensin-converting enzyme inhibitors” as a drug, and wrongly established a relation with “drug-induced pemphigus”. Altogether, false-positive errors accounted for 79% of all errors.

**Table 6:** Error analysis of 100 randomly selected errors on the ADE test set

**Error type**	**Number**
False-positive relations	
Entities correctly identified, with incorrect relation in the knowledge base	64
Entities incorrectly identified, with a relation in the knowledge base	15
False-negative relations	
Entities correctly identified, but relation filtered out	8
Entities not identified, no relation established	13

False-negative errors were generated because the system missed a concept, or did not find a relation in its knowledge base between two correctly found concepts. An example of the first type of error is the term “TMA” (thrombotic microangiopathy), which the system incorrectly recognized as a drug in the sentence “A case report of a patient with probable cisplatin and bleomycin-induced TMA is presented.” The system then missed the relations between the adverse effect “TMA” and the drugs “cisplatin” and “bleomycin”. The other type of false-negative error is illustrated by the sentence “Encephalopathy and seizures induced by intravesical alum irrigations”, which contains two relations, one between “alum” and “encephalopathy”, the other between “alum” and “seizures”. The concept-recognition module found all three concepts correctly, but the knowledge-base module could not find the relation between “alum” and “seizures”. False-negative errors contributed 21% to the total number of errors.

## Discussion

We have investigated the use of NLP and a knowledge base to improve the performance of a system to extract adverse drug events. By applying a set of post-processing rules that utilize POS and chunking information, and exploiting the information contained in the UMLS Metathesaurus and the UMLS Semantic Network, the F-score on the ADE corpus improved by 34.4 percentage points as compared to a simple co-occurrence baseline system. To our knowledge, this is the first study that uses a knowledge base to improve biomedical relation extraction.

The main advantage of our approach as compared to machine-learning approaches is the relatively small set of annotated data required for training. For the ADE corpus, we only used 50 abstracts (3% of the total corpus) to train our system. When we compared our system with a machine-learning system trained on a document set of the same size, our system performed substantially better. Although a machine-learning approach usually performs very well if trained on a sufficiently large training set, the creation of a gold standard corpus (GSC) is tedious and expensive: annotation guidelines have to be established, domain experts must be trained, the annotation process is time-consuming, and annotation disagreements have to be resolved [50]. As a consequence, GSCs in the biomedical domain are generally small and focus on specific subdomains. It should also be noted that even when most of the ADE corpus was used to train the machine-learning system, it did not perform better than our knowledge-based system.

It is difficult to compare the performance of our system with those of the many other relation extraction systems reported in the literature because of the wide variety of relation extraction tasks and evaluation sets. We also evaluated the performance of the whole relation extraction pipeline (similar to, e.g., [51,52]), whereas other studies focused on the relation extraction performance under the assumption that the entities involved were correctly recognized [12,32,53-55]. Moreover, previous systems were sometimes evaluated on a selected set of abstract sentences. As mentioned earlier, Gurulingappa et al. [32] mainly used positive sentences with at least one relation from the abstracts in the ADE corpus, and did not consider relations with nested entities. Similarly, Buyko et al. only used sentences with at least one gene-disease, gene-drug, or drug-disease relation in the PharmGKB database. Both systems obtained F-scores larger than 80%. In a comparable test setting, our system obtained at least as good results (F-score 89%), but in a more realistic test environment, which included the whole relation extraction pipeline and all sentences of the abstracts, performance dropped considerably (F-score 51%). This can largely be attributed to the additional false-positive relations in the negative sentences of the abstracts, decreasing precision considerably. Although our evaluation setting is more realistic, results may still be optimistically biased because our corpus only consisted of abstracts that contain at least one sentence that describes an adverse drug event. The inclusion of abstracts that do not describe adverse drug events would further reduce the system’s precision.

Our error analysis indicated that for the majority of errors the entities are correctly identified (72/100), the error being made in the knowledge-base module. A potential source of false-negative errors is that drugs and adverse events in the knowledge base have no relations with other concepts. However, only 2.8% of the 4700 unique concepts that were found in the ADE corpus did not have any relation. The median number of relations per concept was 22. To reduce the number of false-negative errors, we plan to extend the knowledge base by including relations mined from other drug-adverse effect databases, such as DailyMed [56], DBpedia [57], and DrugBank [58]. False-positive errors generated by the knowledge base may be decreased by including more strict filtering rules on the relation types. We also noted several general concepts, e.g., “patient”, “drug”, and “disease”, that are highly connected. Their removal may improve performance. Finally, we currently took all relation types as equally important and did not consider the plausibility of a path that connects two concepts. Development of a weighting scheme of different relation types and rules that check the plausibility of the possible paths may be able to better distinguish false from true drug-adverse effect relations.

Our system has several limitations. The system currently does not try to distinguish between drug-adverse event relations and drug-disease treatment relations. Further investigation of the relation types in the paths that connect drugs and conditions in the knowledge base may help to differentiate these two situations, but is left for future research. A second limitation is that the knowledge-base module, in order to establish a potential relation, requires concept identifiers as its input. Concept identification is generally considered more difficult than the recognition of named entities, which can serve as the input for machine-learning based relation extraction. Another, related limitation of the current system is that the UMLS Metathesaurus does not provide extensive coverage of genes and proteins. The incorporation of relations from other sources of knowledge, such as UniProt or the databases that are made available through the LODD (Linking Open Drug Data) project, may remedy this drawback.

## Conclusion

We have shown that a knowledge-based approach can be used to extract adverse drug events from biomedical text without need for a large training set. Whether use of a knowledge base is equally advantageous for other biomedical relation extraction tasks remains to be investigated.

## Competing interests

The authors declare that they have no competing interests.

## Authors’ contributions

NK developed the methodology, built the software infrastructure, carried out the experiments, analyzed the data, and drafted the manuscript. BS, BQC and ZA helped to build the software infrastructure. EMM and JAK conceived and supervised the study, co-developed the methodology, and helped to write the paper. All authors read and approved the manuscript.

## References

[B1] Jensen LJ Saric J Bork P Literature mining for the biologist: from information retrieval to biological discovery Nat Rev Genet 2006 7 119 129 10.1038/nrg1768 16418747

[B2] Zweigenbaum P Demner-Fushman D Yu H Cohen KB Frontiers of biomedical text mining: current progress Brief Bioinform 2007 8 358 375 10.1093/bib/bbm045 17977867 PMC2516302

[B3] Simpson MS Demner-Fushman D Aggarwal CC, Zhai C Biomedical text mining: a survey of recent progress Mining Text Data 2012 New York: Springer 465 517

[B4] Revere D Fuller S Characterizing biomedical concept relationships Med Inform (Lond) 2005 8 183 210 10.1007/0-387-25739-X_7

[B5] Dai HJ Chang YC Tzong-Han Tsai R Hsu WL New challenges for biological text-mining in the next decade J Comput Sci Tech 2010 25 169 179 10.1007/s11390-010-9313-5

[B6] Cohen AM Hersh WR A survey of current work in biomedical text mining Brief Bioinform 2005 6 57 71 10.1093/bib/6.1.57 15826357

[B7] Krallinger M Erhardt RAA Valencia A Text-mining approaches in molecular biology and biomedicine Drug Discov Today 2005 10 439 445 15808823 10.1016/S1359-6446(05)03376-3

[B8] Kandula S Zeng-Treitler Q Exploring relations among semantic groups: a comparison of concept co-occurrence in biomedical sources Stud Health Technol Inform 2010 160 995 999 20841833

[B9] Airola A Pyysalo S Björne J Pahikkala T Ginter F Salakoski T All-paths graph kernel for protein-protein interaction extraction with evaluation of cross-corpus learning BMC Bioinformatics 2008 9 S2 19025688 10.1186/1471-2105-9-S11-S2 PMC2586751

[B10] Pyysalo S Airola A Heimonen J Björne J Ginter F Salakoski T Comparative analysis of five protein-protein interaction corpora BMC Bioinformatics 2008 9 S6 18426551 10.1186/1471-2105-9-S3-S6 PMC2349296

[B11] Jang H Lim J Lim J-H Park S-J Lee K-C Park S-H Finding the evidence for protein-protein interactions from PubMed abstracts Bioinformatics 2006 22 e220 e226 10.1093/bioinformatics/btl203 16873475

[B12] Rinaldi F Schneider G Kaljurand K Hess M Andronis C Konstandi O Persidis A Mining of relations between proteins over biomedical scientific literature using a deep-linguistic approach Artif Intell Med 2007 39 127 136 10.1016/j.artmed.2006.08.005 17052900

[B13] Fundel K Küffner R Zimmer R RelEx–relation extraction using dependency parse trees Bioinformatics 2007 23 365 371 10.1093/bioinformatics/btl616 17142812

[B14] Saric J Jensen LJ Ouzounova R Rojas I Bork P Extraction of regulatory gene/protein networks from Medline Bioinformatics 2006 22 645 650 10.1093/bioinformatics/bti597 16046493

[B15] Kang N Van Mulligen EM Kors JA Comparing and combining chunkers of biomedical text J Biomed Inform 2011 44 354 360 10.1016/j.jbi.2010.10.005 21056118

[B16] Huang M Zhu X Li M A hybrid method for relation extraction from biomedical literature Int J Med Inform 2006 75 443 455 10.1016/j.ijmedinf.2005.06.010 16095962

[B17] Buchholz S Marsi E CoNLL-X shared task on multilingual dependency parsing Proceedings of the Tenth Conference on Computational Natural Language Learning; New York, USA 2006 Madison: Omnipress 149 164

[B18] Katrenko S Adriaans P Learning relations from biomedical corpora using dependency tree levels KDECB’06 Proceedings of the 1st International Conference on Knowledge Discovery and Emergent Complexity in Bioinformatics; Ghent, Belgium 2006 Heidelberg: Springer 61 80

[B19] Kim J-H Mitchell A Attwood TK Hilario M Learning to extract relations for protein annotation Bioinformatics 2007 23 256 263 10.1093/bioinformatics/btm168 17646304

[B20] Ozg A Radev DR Semi-supervised classification for extracting protein interaction sentences using dependency parsing Comput Linguist 2007 1 228 237

[B21] Huang Y Lowe HJ Klein D Cucina RJ Improved identification of noun phrases in clinical radiology reports using a high-performance statistical natural language parser augmented with the UMLS specialist lexicon J Am Med Inform Assoc 2005 12 275 285 10.1197/jamia.M1695 15684131 PMC1090458

[B22] Demner-Fushman D Chapman W McDonald C What can natural language processing do for clinical decision support? J Biomed Inform 2009 42 760 772 10.1016/j.jbi.2009.08.007 19683066 PMC2757540

[B23] Hahn U Buyko E Landefeld R Mühlhausen M Poprat M Tomanek K Wermter J An overview of JCoRe, the JULIE lab UIMA component repository Proceedings of the Language Resources and Evaluation Conference (LREC) 2008 Marrakech, Morocco: European Language Resources Association 1 7

[B24] Thorn CF Klein TE Altman RB Pharmacogenomics and bioinformatics: PharmGKB Pharmacogenomics 2010 11 501 505 10.2217/pgs.10.15 20350130 PMC3098752

[B25] Buyko E Beisswanger E Hahn U The extraction of pharmacogenetic and pharmacogenomic relations–a case study using PharmGKB Pac Symp Biocomput; Hawaii, USA 2012 Singapore: World Scientific 376 387 22174293

[B26] Rindflesch TC Fiszman M The interaction of domain knowledge and linguistic structure in natural language processing: interpreting hypernymic propositions in biomedical text J Biomed Inform 2003 36 462 477 10.1016/j.jbi.2003.11.003 14759819

[B27] Aronson AR Effective mapping of biomedical text to the UMLS Metathesaurus: the MetaMap program Proceedings of the AMIA Symposium; Washington, USA 2001 Philadelphia: Hanley & Belfus 17 21 PMC2243666 11825149

[B28] Rindflesch T Fiszman M Libbus B Semantic interpretation for the biomedical research literature Med Inform (Lond) 2005 8 399 422 10.1007/0-387-25739-X_14

[B29] Bodenreider O The Unified Medical Language System (UMLS): integrating biomedical terminology Nucleic Acids Res 2004 32 267 270 10.1093/nar/gkh061 PMC308795 14681409

[B30] Rindflesch TC Aronson AR Kashyap V, Shklar L Semantic processing for enhanced access to biomedical knowledge Real World Semantic Web Applications 2002 Hoboken: John Wiley & Sons 157 172

[B31] Gurulingappa H Fluck J Hofmann-Apitius M Toldo L Identification of adverse drug event assertive sentences in medical case reports First International Workshop on Knowledge Discovery and Health Care Management; Athens, Greece 2011 16 27

[B32] Gurulingappa H Rajput AM Toldo L Extraction of adverse drug effects from medical case reports J Biomed Semantics 2012 3 15 10.1186/2041-1480-3-15 23256479 PMC3599676

[B33] Gurulingappa H Rajput AM Roberts A Fluck J Hofmann-Apitius M Toldo L Development of a benchmark corpus to support the automatic extraction of drug-related adverse effects from medical case reports J Biomed Inform 2012 45 885 892 10.1016/j.jbi.2012.04.008 22554702

[B34] Kano Y Baumgartner WA McCrohon L Ananiadou S Cohen KB Hunter L Tsujii J U-Compare: share and compare text mining tools with UIMA Bioinformatics 2009 25 1997 1998 10.1093/bioinformatics/btp289 19414535 PMC2712335

[B35] Bui QC Sloot PMA A robust approach to extract biomedical events from literature Bioinformatics 2012 28 2654 2661 10.1093/bioinformatics/bts487 22859502

[B36] Tateisi Y Yakushiji A Ohta T Tsujii J Syntax annotation for the GENIA corpus Companion Volume to the Proceedings of the Second International Joint Conference on Natural Language Processing (IJCNLP-05); Jeju Island, Korea 2005 222 227

[B37] Krallinger M Leitner F Rodriguez-Penagos C Valencia A Overview of the protein-protein interaction annotation extraction task of BioCreative II Genome Biol 2008 9 S4 18834495 10.1186/gb-2008-9-s2-s4 PMC2559988

[B38] Leitner F Mardis SA Krallinger M Cesareni G Hirschman LA Valencia A An overview of BioCreative II. 5 Comput Biol Bioinform 2010 7 385 399 10.1109/tcbb.2010.61 20704011

[B39] Krallinger M Vazquez M Leitner F Salgado D Chatr-aryamontri A Winter A Perfetto L Briganti L Licata L Iannuccelli M The protein-protein interaction tasks of BioCreative III: classification/ranking of articles and linking bio-ontology concepts to full text BMC Bioinformatics 2011 12 S3 22151929 10.1186/1471-2105-12-S8-S3 PMC3269938

[B40] Kim J-D Ohta T Pyysalo S Kano Y Tsujii J Overview of BioNLP’09 shared task on event extraction Proceedings of the Workshop on BioNLP Shared Task; Boulder, USA 2009 Madison: Omnipress 1 9

[B41] Kim JD Pyysalo S Ohta T Bossy R Nguyen N Tsujii J Overview of BioNLP shared task 2011 Proceedings of the BioNLP Shared Task 2011 Workshop; Portland, USA 2011 Madison: Omnipress 1 6

[B42] Rinaldi F Clematide S Garten Y Whirl-Carrillo M Gong L Hebert JM Sangkuhl K Thorn CF Klein TE Altman RB Using ODIN for a PharmGKB revalidation experiment Database J Biol Database Curr 2012 2012 bas021 10.1093/database/bas021 PMC3332569 22529178

[B43] Ferrucci D Lally A UIMA: an architectural approach to unstructured information processing in the corporate research environment Nat Lang Eng 2004 10 327 348 10.1017/S1351324904003523

[B44] Schuemie MJ Jelier R Kors JA Peregrine: lightweight gene name normalization by dictionary lookup Proceedings of the BioCreAtIvE II Workshop; Madrid, Spain 2007 131 133

[B45] Bodenreider O McCray AT Exploring semantic groups through visual approaches J Biomed Inform 2003 36 414 432 10.1016/j.jbi.2003.11.002 14759816 PMC1997308

[B46] Hettne KM Van Mulligen EM Schuemie MJ Schijvenaars BJ Kors JA Rewriting and suppressing UMLS terms for improved biomedical term identification J Biomed Semantics 2010 1 1 5 10.1186/2041-1480-1-1 20618981 PMC2895736

[B47] Kang N Singh B Afzal Z van Mulligen EM Kors JA Using rule-based natural language processing to improve disease normalization in biomedical text J Am Med Inform Assoc 2012 doi: 10.1136/amiajnl–2012–001173 10.1136/amiajnl-2012-001173 PMC3756254 23043124

[B48] Schwartz Hearst MA AS: a simple algorithm for identifying abbreviation definitions in biomedical text Proceedings of the 8th Pacific Symposium on Biocomputing; Hawaii, USA 2003 Singapore: World Scientific 451 462 12603049

[B49] Hanisch D Fundel K Mevissen H-T Zimmer R Fluck J ProMiner: rule-based protein and gene entity recognition BMC Bioinformatics 2005 6 S14 15960826 10.1186/1471-2105-6-S1-S14 PMC1869006

[B50] Kang N van Mulligen EM Kors JA Training text chunkers on a silver standard corpus: can silver replace gold? BMC Bioinformatics 2012 30 13 10.1186/1471-2105-13-17 PMC3280170 22289351

[B51] Bundschus M Dejori M Stetter M Tresp V Kriegel HP Extraction of semantic biomedical relations from text using conditional random fields BMC Bioinformatics 2008 9 207 10.1186/1471-2105-9-207 18433469 PMC2386138

[B52] Islamaj Doğan R Névéol A Lu Z A context-blocks model for identifying clinical relationships in patient records BMC Bioinformatics 2011 12 Suppl 3 S3 10.1186/1471-2105-12-S3-S3 21658290 PMC3111589

[B53] Melton GB Hripcsak G Automated detection of adverse events using natural language processing of discharge summaries J Am Med Inform Assoc 2005 12 448 457 10.1197/jamia.M1794 15802475 PMC1174890

[B54] Chun HW Tsuruoka Y Kim JD Shiba R Nagata N Hishiki T Tsujii J Extraction of gene-disease relations from Medline using domain dictionaries and machine learning Pac Symp Biocomput; Hawaii, USA 2006 Singapore: World Scientific 4 15 17094223

[B55] Uzuner O South BR Shen S Duvall SL i2b2/VA challenge on concepts, assertions, and relations in clinical text J Am Med Inform Assoc 2010 2011 18 552 556 10.1136/amiajnl-2011-000203 PMC3168320 21685143

[B56] Elkin PL Carter JS Nabar M Tuttle M Lincoln M Brown SH Drug knowledge expressed as computable semantic triples Stud Health Technol Inform 2011 166 38 47 21685609

[B57] Bizer C Lehmann J Kobilarov G Auer S Becker C Cyganiak R Hellmann S DBpedia–a crystallization point for the web of data Web Seman Scie Serv Age WWW 2009 7 154 165 10.1016/j.websem.2009.07.002

[B58] Wishart DS Knox C Guo AC Shrivastava S Hassanali M Stothard P Chang Z Woolsey J DrugBank: a comprehensive resource for in silico drug discovery and exploration Nucleic Acids Res 2006 34 668 672 10.1093/nar/gkj067 PMC1347430 16381955

